# pH-Triggered Controlled Release of Chlorhexidine Using Chitosan-Coated Titanium Silica Composite for Dental Infection Prevention

**DOI:** 10.3390/pharmaceutics16030377

**Published:** 2024-03-08

**Authors:** Mrinal Gaurav Srivastava, Nur Hidayatul Nazirah Kamarudin, Merve Kübra Aktan, Kai Zheng, Naiera Zayed, Derick Yongabi, Patrick Wagner, Wim Teughels, Aldo R. Boccaccini, Annabel Braem

**Affiliations:** 1 KU Leuven, Department of Materials Engineering (MTM), 3001 Leuven, Belgium; mrinalgaurav.srivastava@kuleuven.be (M.G.S.); nhnazirah@ukm.edu.my (N.H.N.K.); mervekubra.aktan@kuleuven.be (M.K.A.); 2 Universiti Kebangsaan Malaysia, Department of Chemical and Process Engineering, Faculty of Engineering and Built Environment, Bangi 43600, Malaysia; 3 University of Erlangen-Nuremberg, Department of Materials Science and Engineering, Institute of Biomaterials, 91058 Erlangen, Germany; kaizheng@njmu.edu.cn (K.Z.); aldo.boccaccini@fau.de (A.R.B.); 4 Nanjing Medical University, Engineering Research Center of Stomatological Translational Medicine, Nanjing 210029, China; 5 KU Leuven, Department of Oral Health Sciences, 3000 Leuven, Belgium; naiera.zayed@kuleuven.be (N.Z.); wim.teughels@kuleuven.be (W.T.); 6 Ghent University, Department of Biotechnology, Center for Microbial Ecology and Technology (CMET), 9000 Gent, Belgium; 7 KU Leuven, Department of Physics and Astronomy, Laboratory for Soft Matter and Biophysics, 3001 Leuven, Belgium; derick.yongabi@kuleuven.be (D.Y.); patrickhermann.wagner@kuleuven.be (P.W.); 8 University Hospitals Leuven, Dentistry, 3000 Leuven, Belgium

**Keywords:** controlled drug release, pH response, chitosan, mesoporous silica, titanium, chlorhexidine, biofilm inhibition, dental implant

## Abstract

Peri-implantitis is a growing pathological concern for dental implants which aggravates the occurrence of revision surgeries. This increases the burden on both hospitals and the patients themselves. Research is now focused on the development of materials and accompanying implants designed to resist biofilm formation. To enhance this endeavor, a smart method of biofilm inhibition coupled with limiting toxicity to the host cells is crucial. Therefore, this research aims to establish a proof-of-concept for the pH-triggered release of chlorhexidine (CHX), an antiseptic commonly used in mouth rinses, from a titanium (Ti) substrate to inhibit biofilm formation on its surface. To this end, a macroporous Ti matrix is filled with mesoporous silica (together referred to as Ti/SiO_2_), which acts as a diffusion barrier for CHX from the CHX feed side to the release side. To limit release to acidic conditions, the release side of Ti/SiO_2_ is coated with crosslinked chitosan (CS), a pH-responsive and antimicrobial natural polymer. Scanning electron microscopy coupled with energy dispersive X-ray spectroscopy (SEM/EDX) and Fourier transform infrared (FTIR) spectroscopy confirmed successful CS film formation and crosslinking on the Ti/SiO_2_ disks. The presence of the CS coating reduced CHX release by 33% as compared to non-coated Ti/SiO_2_ disks, thus reducing the antiseptic exposure to the environment in normal conditions. Simultaneous differential scanning calorimetry and thermogravimetric analyzer (SDT) results highlighted the thermal stability of the crosslinked CS films. Quartz crystal microbalance with dissipation monitoring (QCM-D) indicated a clear pH response for crosslinked CS coatings in an acidic medium. This pH response also influenced CHX release through a Ti/SiO_2_/CS disk where the CHX release was higher than the average trend in the neutral medium. Finally, the antimicrobial study revealed a significant reduction in biofilm formation for the CS-coated samples compared to the control sample using viability quantitative polymerase chain reaction (v-qPCR) measurements, which were also corroborated using SEM imaging. Overall, this study investigates the smart triggered release of pharmaceutical agents aimed at inhibiting biofilm formation, with potential applicability to implant-like structures.

## 1. Introduction

Statistics show that over the past years, the demand for dental implants has increased significantly [1]. This is mainly because of the aging population, people suffering from trauma and even for aesthetic purposes [2]. As the usage is increasing, cases concerning infection development are also rising, which is a matter of concern for both the patients as well as the healthcare system. Compared to infection in orthopedic implants like primary hip and knee replacements, which is approximately 1% and increases to around 2–5% for revision surgeries, the occurrence of peri-implantitis in dental implants has risen to values ranging from 10 to 22% [3,4,5]. This difference is because of hostile oral environments consisting of different dietary habits and direct exposure to various biofilm-forming micro-organisms, i.e., pathogens. Biofilm is a slimy protective extracellular matrix made out of extracellular polymeric substances produced by pathogens adhering to the dental implant surface, especially in periodontal pockets, which are the gaps between the implant and the surrounding soft tissues [6,7]. Development of biofilm can cause conditions of infection (i.e., peri-implant mucositis) followed by inflammation of the peri-implant tissues (i.e., peri-implantitis) which can lead to bone loss and in severe cases implant failure [8]. Surgical interventions are often needed in the case of established biofilms. This can be avoided by developing implants that can resist the growth of pathogenic biofilms by using physical/chemical material treatment strategies and can also be coupled with incorporating antimicrobial agents on the material surface [9]. Such materials can be termed anti-biofouling (preventing or resisting attachment of microorganisms) and bactericidal (contact killing of microorganisms) [10]. These materials are developed by incorporating passive (anti-adhesive materials) or active (antimicrobial-releasing) coatings and also by modifying the surface topography/chemistry of the implant [11,12]. Although these recent developments are very innovative, they have the shortcomings of the coating being fragile against mechanical loading inside the oral environment, the short duration of antimicrobial release after implantation with therapeutic concentrations and the modified surface topography becoming less effective due to an accumulation of absorbed surface conditioning on the film.

To overcome this limitation, a methodology was developed in our research group to incorporate release-controlling material in a protective matrix and establish long-term release from a refillable reservoir. To this end, previously, a Ti/SiO_2_ composite disk formed by silica particle injection into a macroporous Ti disk was successfully developed as an antimicrobial drug-release medium where Ti was a load-bearing structure and the silica acted as a diffusion barrier for the drug. Braem et al., 2015, demonstrated the ability of controlled release of an antibiofilm compound, toremifene, through a mesoporous silica diffusion barrier filled inside macroporous Ti disks with interconnected porosity to significantly reduce the growth of *C. albicans* biofilm formation [13]. This approach was stepped up by De Cremer et al., 2017, where a dental implant was manufactured and had a similar structural composition of a macroporous Ti disk filled with mesoporous silica, acting as a diffusion barrier for an anti-infective agent called chlorhexidine [14]. Antimicrobial tests using *S. mutans* as the bacterial strain indicated almost complete prevention of the growth of the biofilm but cell culture testing using alveolar osteoblasts showed some degree of cytotoxicity above 1 µM concentration.

It is important to control the amount and period of drug release to reduce cytotoxicity to the host cells, and having a capping agent on top of the silica-infiltrated macropores of Ti is of interest [15,16]. Since some biofilms create an acidic environment around them, chitosan (CS), due to its excellent film-forming ability and inherent pH-responsive and antimicrobial properties, is a promising material for this role [17,18]. CS is a natural polysaccharide and has been abundantly used in biomedical applications as a drug-delivery agent [19,20,21,22]. Due to its ability to crosslink, it has also been used to form robust films on Ti substrates by crosslinking with silica precursors like tetraethoxysilane (TEOS) and 3-glycydoxytrimethoxysilane (GPTMS), and it releases silver nanoparticles, which increases its antimicrobial properties [23,24]. Apart from coatings, CS is also known to form nanoparticles, capsules and hydrogels [22,25]. Dip coating, drop coating, spin coating, electrophoretic deposition, etc. are common methods for forming CS coatings on substrates [26,27].

In this work, we will investigate for the first time the drug-release rate and antibacterial efficacy of a GPTMS-crosslinked CS-capped Ti/SiO_2_ composite substrate. The synergy between the CS and the Ti/SiO_2_ sample will be investigated by using different wt% of CS coating solution, which will be used to study the influence on CHX release. As a proof-of-concept, the combined antimicrobial efficacy of the CS coating and CHX diffusion through the GPTMS-crosslinked CS-capped Ti/SiO_2_ composite substrate is evaluated using the oral bacterial cariogenic pathogen *S. sobrinus* and periopathogen *F. nucleatum*.

## 2. Materials and Methods

### 2.1. Fabrication of Ti/SiO_2_/CS Disk and CS Films

Macroporous Ti base substrates were manufactured using the procedure described by Braem et al., 2015 [13]. Briefly, Ti/TiH_2_ powder mixture was prepared by mixing Ti (grade S < 8 µm, min. 98.7% Ti, Albermarle, Langelsheim, Germany) and TiH_2_ (grade VM, min. 94% Ti and min. 3.7% H, Albermarle, Langelsheim, Germany) powder in a 90:10 molar ratio for 24 h in an Ar atmosphere using a multidirectional mixer (WAB Turbula T2A, Muttenz, Switzerland). A hardened steel die was used to press 0.5 g of the prepared powder mixture at 50 kN for 1 min. The produced powder pellets (Ø = 12.2 mm, h = 1.3 mm) were stepwise sintered for complete dehydrogenation using a vacuum furnace (Brew TTDL HT Vac) at 10^−6^ mbar pressure. The stepwise sintering started by increasing the temperature to 450 °C at a heating rate of 5 °C/min and a dwell time of 1 h. The temperature was then raised to 550 °C at a heating rate of 2 °C/min and a dwell time of 1 h, followed by heating to 650 °C using a heating rate and dwell time similar to those in the previous step. Finally, the temperature was increased to 850 °C at 10 °C/min with a dwell time of 5 min. The disks were cooled down passively inside the furnace after this heating cycle. Through this process, partially sintered and consolidated disks were obtained.

The preparation of silica sol was adapted from the procedure described by Braem et al., 2015 [13]. Briefly, commercially available colloidal silica sol in water (Ludox^®^ HS-40, Sigma-Aldrich, Steinheim, Germany) and 0.055 M HCl (37% Chemlab, Zedelgem, Belgium) solution in ultrapure water (Milli-Q, 18.2 MΩcm, Merck Millipore, Darmstadt, Germany) were mixed in a 1:1 volume ratio and magnetically stirred for 10 min. The produced silica sol was injected into macroporous Ti disks following the approach illustrated by Braem et al., 2015, with modifications [13]. A syringe pump (NE-1000, New Era, Farmingdale, NY, USA) was used for injection instead of a high-pressure pump. Silica sol was filled inside a syringe (Ø = 15 mm, Inject^®^ Luer Lock Solo, Braun, Diegem, Belgium), which was connected using silicone tubes (1.5 mm (ID) × 3.75 mm (OD), Saint-Gobain, Courbevoie, France) to an in-house-developed injection cell, with the Ti disk inside, and injection was carried out at a flow rate of 2 mL/min. The injected disk was dried overnight at 60 °C (Binder, VWR, Tuttlingen, Germany) followed by re-injection and re-drying, as carried out previously. After two injection and drying steps, the final stage was the calcination of the silica-injected disks by heating at a rate of 1 °C/min to 225 °C (Nabertherm L5-12, Lilienthal, Germany) followed by a 6 h dwell time.

A chitosan (CS) solution of 1 wt% was prepared by dissolving medium molecular weight CS powder (75–85% deacetylated chitin, Sigma-Aldrich, Steinheim, Germany) in a 2 vol% acetic acid (100%, Merck, Darmstadt, Germany) solution at 55 °C for 24 h following the procedure developed by Gan et al., 2015 [28]. The obtained solution was crosslinked with 15 vol% of (3-glycidyloxipropyl) trimethoxy-silane (GPTMS, >98%, Sigma-Aldrich, Steinheim, Germany), which was prepared by dilution in absolute ethanol (>99.5%, AnalaR NOMAPURE, Fontenay-sous-Bois, France). An aliquot of 0.5 mL from this solution was mixed with 20 mL of the previously obtained CS solution and mixed in a plastic container [28]. The solution was kept stable at room temperature for 1 day to react/couple. The crosslinked 1 wt% CS solution (CS1/G) was diluted to 0.5 wt% (CS0.5/G) and 0.1 wt% (CS0.1/G). Ti/SiO_2_/CS samples were prepared by dropping three drops of the respective CS solution from a Pasteur pipette onto the surface of the disks, followed by drying them inside a fume hood for 48 h.

For in-depth CS film analysis regarding swelling in different media and the effect of crosslinking, free-standing CS films were formed via solvent casting and were spin-coated on a crystal for quartz crystal microbalance with dissipation monitoring (QCM-D) measurements. To form free-standing films, 15 mL of the non-crosslinked CS and 1CS/G solution was put in different Petri dishes and allowed to dry inside a fume hood for 48 h. The dried films were peeled off and used for further experiments. AT-cut quartz crystals (14 mm diameter, 0.3 mm thickness, 5 MHz resonance frequency; Biolin Scientific, Gothenburg, Sweden) with gold coating (50 nm thickness) were spin-coated with CS1/G solution. The protocol recommended by the manufacturer was followed to clean the crystals. The crystals were dried inside a fume hood for 48 h after coating.

### 2.2. Characterization of Ti/SiO_2_/CS Disk and CS Films

The methodology to analyze the degree of silica filling inside the macroporous Ti disk was adopted from Braem et al. [13]. Airflow measurements were conducted using a bubble flow meter (Scantec, Antwerp, Belgium). Briefly, compressed air at 3 bar was allowed to pass through the Ti/SiO_2_ disk fixed inside a Luer-lock, and the rate at which the air bubble rose inside the glass burette of the flow meter attached on the other end indicated the silica filling degree. 

The presence of CS coating on the Ti/SiO_2_/CS disk was verified using Fourier transform infrared (FT-IR) spectroscopy with the attenuated total reflectance (ATR) accessory (Vertex 70, Bruker, Ettlingen, Germany). FT-IR was also used to study the formation of a crosslinked network in GPTMS-crosslinked CS. Scans were carried out in the wavenumber range from 400–4000 cm^−1^ at a resolution of 4 cm^−1^ and 64 scans per measurement. A scanning electron microscope (SEM, XL-30, FEG, FEI, Eindhoven, The Netherlands) was used at 10 kV throughout the study. Semi-quantitative composition analysis was conducted using SEM (Nova NanoSEM 450, FEI, Eindhoven, The Netherlands) with associated energy dispersive X-ray spectroscopy (EDX, EDAX). Before imaging, the surfaces were coated with a 5 nm-thick Pt/Pd coating (Q150TS, Quorum, Kent, UK) to prevent charging. Simultaneous differential scanning calorimetry (DSC) and a thermogravimetric analyzer (TGA) (SDT Q600, TA Instruments, New Castle, DE, USA) at a 10 °C/min ramp rate under a nitrogen environment was used to study the thermal stability of the non-crosslinked and crosslinked CS films.

Swelling measurements were performed using non-crosslinked CS films and GPTMS-crosslinked CS films in pH-neutral water to study the effect of crosslinking on the swelling behavior of CS films. In both studies, dried films were immersed in an aqueous solution for certain time intervals. Excess water was removed from their surface by blotting the films with a paper cloth and then weighing them until a constant value was achieved. The swelling % was calculated using Equation (1).

(1) $$Swelling\%=\frac{Weight\;of\;wet\;film-Weight\;of\;dry\;film}{Weight\;of\;dry\;film}\times 100$$

QCM-D measurements were performed using a Q-sense instrument (Biolin Scientific, Gothenburg, Sweden) on 1CS/G-coated crystals to study the effect of pH on the swelling characteristics of the CS films in situ. Neutral and acidic (pH 4.5 by dropwise adding 0.1 M HCl) Milli-Q water was pumped through the flow chambers at a flow rate of 0.5 µL/min. The frequency shift (Δf) and changes in energy dissipation (ΔD) were recorded as a function of time at room temperature.

### 2.3. Drug-Release Studies

**Figure 1:**
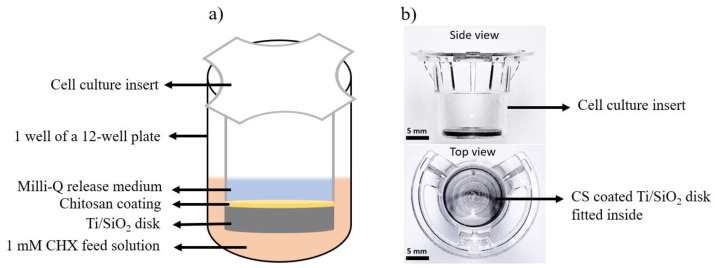
(**a**) Schematic of the drug-release setup with CS-coated Ti/SiO_2_ disk fitted inside the cell culture insert with the Milli-Q release medium and CHX feed medium on both sides of the disk. (**b**) Actual cell culture insert with a CS-coated Ti/SiO_2_ disk fitted inside.

Drug-release experiments were conducted using cell culture inserts similar to those used by Braem et al. [13]. Chlorhexidine diacetate salt hydrate (CHX, Sigma-Aldrich) of 0.1 mM concentration was used as the feed medium. To ensure that the feed medium and the release medium were at the same height level, the cell culture inserts had 1.5 mL of feed solution and 0.5 mL of release medium separated by the Ti/SiO_2_/CS disk. This setup allowed drug diffusion to occur only via the concentration gradient between the two sides of the disk and negated factors such as height difference, which can act as an extra driving force for drug diffusion. A schematic of the drug-release setup is shown in Figure 1a, and the actual disk fitted inside the cell culture insert is shown in Figure 1b. Aliquots of the release medium were collected at fixed time intervals, and ultraviolet-visible (UV-Vis, Varioskan, VWR, Leuven, Belgium) spectroscopy was used to measure the absorbance of CHX, which was used as an anti-infective agent, thus quantifying the amount of drug released over the given period. pH-neutral Milli-Q water and acidic Milli-Q water were used as the release medium and were regularly renewed to mimic in vivo conditions. Maximum CHX absorbance was obtained at 255 nm and this wavelength was used to plot the calibration curves for the concentration of the CHX ranging from 6.255 µg/mL to 31.275 µg/mL in neutral Milli-Q water (R^2^ = 0.987) and in acidic Milli-Q water (R^2^ = 0.993).

### 2.4. Antimicrobial Study

Two-species biofilms were grown on the surface of the tested discs. The biofilms encompassed one biofilm-forming species, *Streptococcus sobrinus* (ATCC 20742), and one periodontal pathogen, *Fusobacterium nucleatum* (ATCC 20482). The bacterial species were maintained on blood agar (Oxoid LTD, Basingstoke, Hampshire, UK) supplemented with 5 mg/L hemin (Merk Life Sciences U.K. LTD, Gillingham, UK), 1 mg/L menadione (Merk Life Sciences U.K. LTD, Gillingham, U.K.) and 5% sterile horse blood (E&O Laboratories, Edinburgh, Scotland), under aerobic (5% CO_2_) or anaerobic (80% N_2_, 10% H_2_ and 10% CO_2_) conditions for *S. sobrinus* and *F. nucleatum*, respectively. Overnight, liquid cultures were prepared in brain heart infusion (BHI) broth (Difco) for *S. sobrinus*, and BHI supplemented with 0.04% L-Cysteine HCl (BHIC) for *F. nucleatum*, under the same specific incubation conditions. The overnight cultures were then adjusted to concentrations of 10^8^ events/mL each using flow cytometry (FACSVerse cytometer, BD Biosciences, Erembodegem, Belgium) with a 488 nm blue and a 640 nm red laser [29]. These cultures were used to grow biofilms on the tested surfaces in Brain Hearth Infusion 2 (BHI-2) broth containing BHI supplemented with 2.5 g/L mucin, 1.0 g/L yeast extract, 0.1 g/L cysteine, 2.0 g/L sodium bicarbonate and 0.25% (*v*/*v*) glutamic acid. The biofilms were allowed to grow for 24 h under microaerophilic conditions (6% O_2_, 7% CO_2_, 7% H_2_ and 80% N_2_) while shaking at 100 rpm. After that, the biofilms were collected, and bacterial DNA extractions and v-qPCR were performed, according to Zayed et al. [29].

## 3. Results

### 3.1. Characterization of Ti/SiO_2_/CS Disks and CS Films

**Figure 2:**
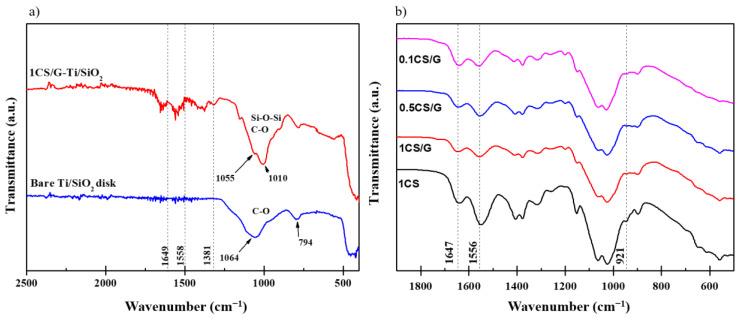
FT-IR spectra (**a**) confirming the successful functionalization of Ti/SiO_2_ disk with GPTMS-crosslinked CS solution indicated by amide I and amide II bands at wavenumbers 1649 and 1558 cm^−1^ and (**b**) comparison of free-standing films of 1, 0.5 and 0.1 wt% CS crosslinked with GPTMS indicating crosslinking through the primary amine group at 1556 cm^−1^.

**Figure 3:**
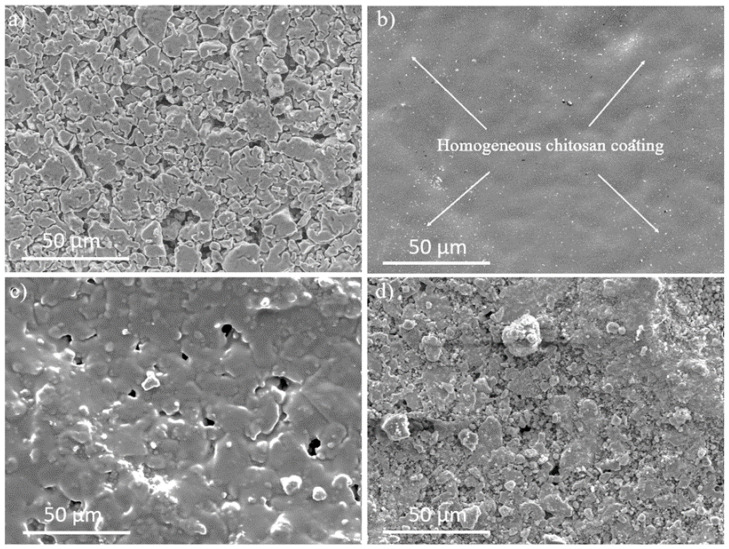
Representative SEM micrographs comparing (**a**) non-coated Ti/SiO_2_ disks and (**b**) 1 wt% CS-coated Ti/SiO_2_ disks indicating homogeneous chitosan distribution highlighted using white arrows. (**c**) Shows a fragmented 0.5 wt% CS coating and (**d**) shows that 0.1 wt% CS coating barely covers the surface.

Ti/SiO_2_ disks with an airflow rate of below 100 mL/min were selected for experiments to ensure comparable silica filling inside the macroporous Ti disks. Macroporous Ti disks without SiO_2_ filling had an airflow rate of more than 1000 mL/min. The airflow through the Ti/SiO_2_/CS disks was almost negligible. FT-IR spectroscopy (Figure 2) and SEM (Figure 3) were used to verify the CS film on the surface of the Ti/SiO_2_/CS composite disk. 

Figure 2a confirms the successful functionalization of the Ti/SiO_2_ disk with CS due to the formation of bands at 1649 and 1558 cm^−1^ corresponding to amide I and amide II groups, respectively, which are present in CS monomers [30]. Due to the background generated on solid samples, FT-IR measurements were also conducted on crosslinked and non-crosslinked CS free-standing films to confirm the coupling reaction between GPTMS and CS during the preparation of the GPTMS-crosslinked CS films. Figure 2b is a comparison of the spectra indicating the presence of an epoxide peak at 921 cm^−1^ confirming GPTMS incorporation into the crosslinked CS films [23]. The reduction in the relative intensity of the primary amine peak at 1556 cm^−1^ in the crosslinked CS film compared to the non-crosslinked CS film indicates that GPTMS coupling takes place through the primary amine [23]. This reduction is not observed in the secondary amine peak at 1635 cm^−1^ [23]. This indicates that the secondary amine group does not take part in the coupling of CS with GPTMS. SEM imaging of the top surface of uncoated Ti/SiO_2_ and Ti/SiO_2_/CS disks with different CS coating compositions (CS1/G, CS0.5/G and CS0.1/G) is presented in Figure 3. The CS1/G-coated Ti/SiO_2_ disk SEM micrograph (Figure 3b) shows a uniform CS coating on its surface compared to the uncoated Ti/SiO_2_ disk (Figure 3a), which is not visible in the prior sample. CS0.5/G (Figure 3c) has a fragmented coating structure, while CS0.1/G (Figure 3d) is not sufficient to form a coating. CS0.1/G was therefore eliminated from further evaluations.

**Figure 4:**
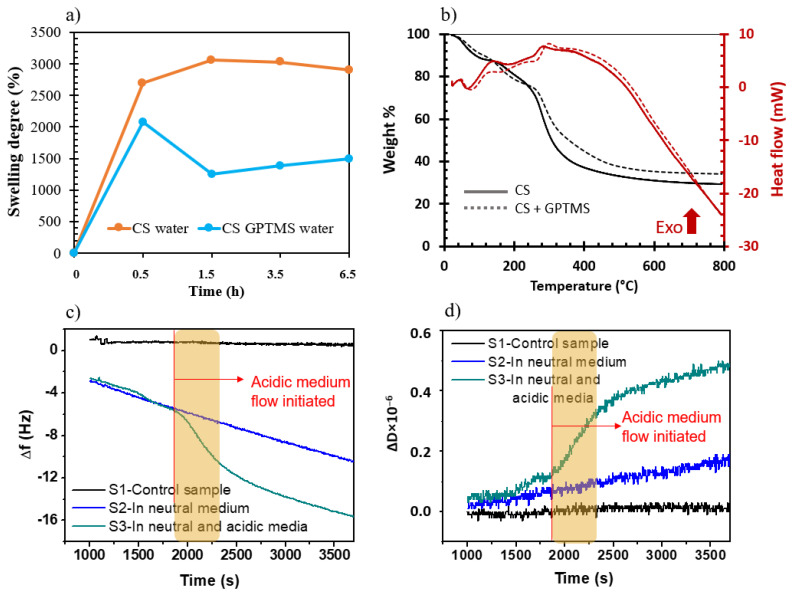
Results comparing the swelling characteristics of (**a**) non-crosslinked CS film and GPTMS-crosslinked CS film in a neutral medium. Non-crosslinked CS film has almost double the swelling % compared to GPTMS-crosslinked CS film. (**b**) SDT measurements indicate better thermal stability for crosslinked films than non-crosslinked CS films. QCM-D measurements highlight the time-dependent changes in (**c**) the frequency shift (Δf) and (**d**) energy dissipation (ΔD) with the introduction of an acidic medium. These changes in Δf and ΔD indicate a higher rate and amount of water adsorption and swelling for 1CS/G film in an acidic medium than in a neutral medium.

Swelling and thermal stability tests (Figure 4) were conducted to study the pH response and effect of GPTMS on the free-standing CS film.

Figure 4a confirms that the non-crosslinked CS film has almost double the swelling percentage compared to the GPTMS-crosslinked CS film. The swelling percentage became constant after a couple of hours of immersion of the film in the testing medium. Since it has been reported that non-crosslinked CS films tend to disintegrate and dissolve in an acidic medium, swelling tests were not conducted on them [31]. SDT signals in Figure 4b indicate earlier weight loss and higher heat evolution for the non-crosslinked CS film than the crosslinked CS film until 140 °C. After 140 °C, the TGA signal drops for the crosslinked CS film, following the trend of the non-crosslinked CS film, and DSC signals of the crosslinked CS approach the non-crosslinked CS. After 240 °C, the non-crosslinked CS film undergoes a sharper weight loss compared to the crosslinked CS film and higher heat evolves for the crosslinked CS film than the non-crosslinked CS film after 290 °C. To study the influence of pH change on the swelling behavior of CS films, QCM-D measurements were performed. Three conditions were tested: The first sample was a control sample of uncoated crystal (S1) tested first in a neutral medium and then in an acidic medium to negate the influence of the crystal itself. The second sample was a 1CS/G-coated crystal tested only in neutral Milli-Q water (S2) to decouple the influence of neutral medium, and the third sample was a 1CS/G-coated crystal first in neutral medium and then in acidic medium (S3) to study the effect of pH change on swelling. In Figure 4c,d a clear pH response is observed for the 1CS/G-coated crystal in an acidic medium (S3) highlighted in the orange region where, in about 10 min, Δf increases from −5 Hz to −15 Hz before re-establishing a linear trend. As expected, the corresponding energy dissipation factor, ΔD, also displays an increase of approximately 0.4 × 10^−6^. In comparison with S2, which was tested continuously in a neutral medium and had a linear trend, the onset of the acidic medium had a clear deviation from the linear trend for S3. The constant Δf and ΔD with time for S1 indicate that the inflow of neutral and acidic media did not affect the uncoated crystal.

### 3.2. Chlorhexidine-Release Study

**Figure 5:**
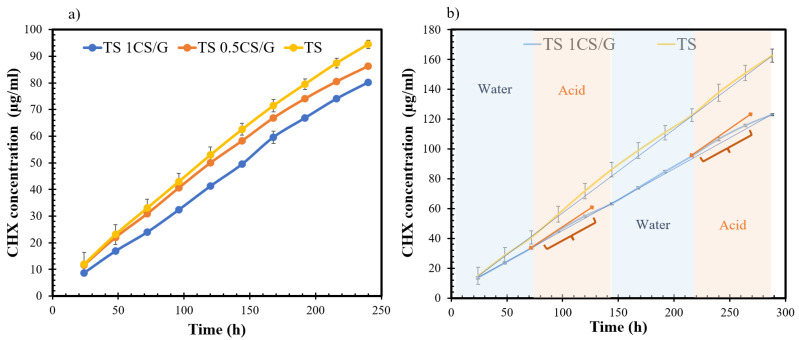
CHX-release studies through Ti/SiO_2_/CS disks in different experimental conditions: (**a**) comparison of CHX-release profiles of non-coated Ti/SiO_2_ disks with 1 wt% and 0.5 wt% CS-coated Ti/SiO_2_/CS disks in neutral release medium. Ti/SiO_2_ disk was a reference for all the release studies and it can be observed that the CHX release was significantly suppressed for higher CS wt% Ti/SiO_2_-coated disks. (**b**) Comparison of CHX-release profiles in alternating neutral and acidic release media with the red line (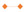) of higher slope indicating an increased release in the acidic regions for CS-coated disk highlighted using curly brackets.

Diffusion of CHX through Ti/SiO_2_/CS was studied using UV–Vis spectroscopy, and a comparison was made between the amount of CHX released through the Ti/SiO_2_ and Ti/SiO_2_/CS disks. From Figure 5a, it can be observed that CHX release in the neutral Milli-Q water medium was detected the following day in all the samples after insertion of the feed medium. The presence of a higher wt% CS coating limited the CHX release over the tested duration of ten days. The daily CHX release rate is in the range of 68.22 µg/mL (0.109 mM) and 97.49 µg/mL (0.155 mM) for the 1CS/G- and 0.5CS/G-coated Ti/SiO_2_ disks, respectively, and 101.69 µg/mL (0.162 mM) for the uncoated Ti/SiO_2_ disk. The release rate of CHX is lowest in the case of 1 wt% CS-coated Ti/SiO_2_ disks followed by the 0.5 wt% CS coating. Overall, the drug-release profiles indicate that CHX release is dependent on the CS wt% in the coating composition with the release being suppressed for higher CS wt%. Results showing CHX release in alternating neutral and acidic Milli-Q water media shown in Figure 5b indicate an increase in the CHX release when the release medium was acidic. This increase is evident from the increase in slope in the acidic regions compared to the original slope observed in the trendline drawn across release points within the neutral medium and highlighted using curly brackets. The slope of the Ti/SiO_2_ release curve passes through the range of standard deviation, which implies that the CHX release was not affected by the change in release medium. This pH-responsive nature of 1CS/G film is more clearly observed in the QCM-D study performed in this research.

**Figure 6:**
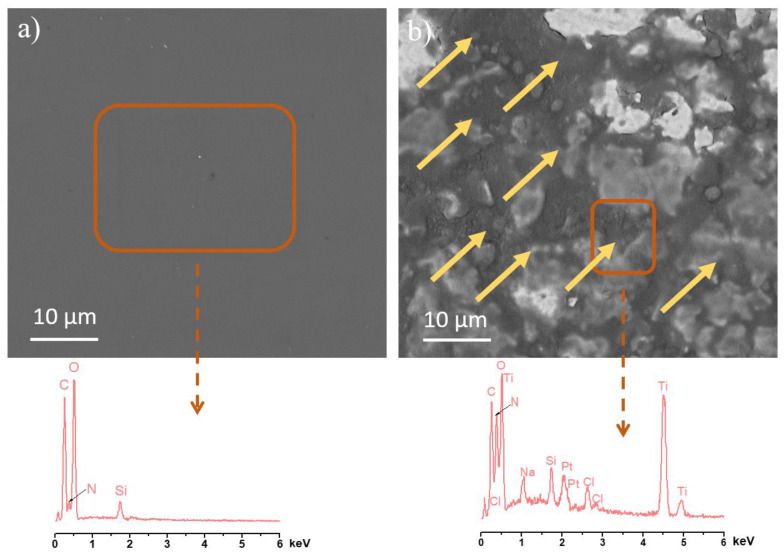
SEM/EDX data semi-quantitatively comparing the 1CS/G mass coated on Ti/SiO_2_/CS (**a**) before and (**b**) after the CHX-release study. Organic chitosan peaks are present for both conditions with additional background coming from Ti and SiO_2_ in (**b**). Yellow arrows (→) in (**b**) indicate CS over the macropores of Ti filled with silica. Presence of CS as a capping agent over these regions leads to suppressed CHX release. Orange box and dashed arrows indicate the area scanned for EDX analysis.

The SEM/EDX results shown in Figure 6 are a comparison of the Ti/SiO_2_/CS sample before and after the CHX-release study. EDX data in Figure 6a semi-quantitatively represent the organic chitosan coating with no signals from the underlying Ti substrate. EDX data in Figure 6b have the same organic peaks semi-quantitatively indicating the presence of a CS coating but Ti and SiO_2_ peaks have also appeared, indicating that the mass of the 1CS/G coating on the Ti/SiO_2_ disk after immersing it in an aqueous medium reduces where CS is restricted to the macropores of Ti containing silica nanoparticles. This implies that the suppressed CHX release is not because of the entire CS coating, which was deposited before the CHX release testing, but due to these CS regions covering the silica inside the macropores of Ti. Na and Cl peaks correspond to the salts from the PBS release medium and the Pt peak corresponds to the Pt/Pd coating carried out before SEM/EDX measurement.

### 3.3. Antimicrobial Activity

**Figure 7:**
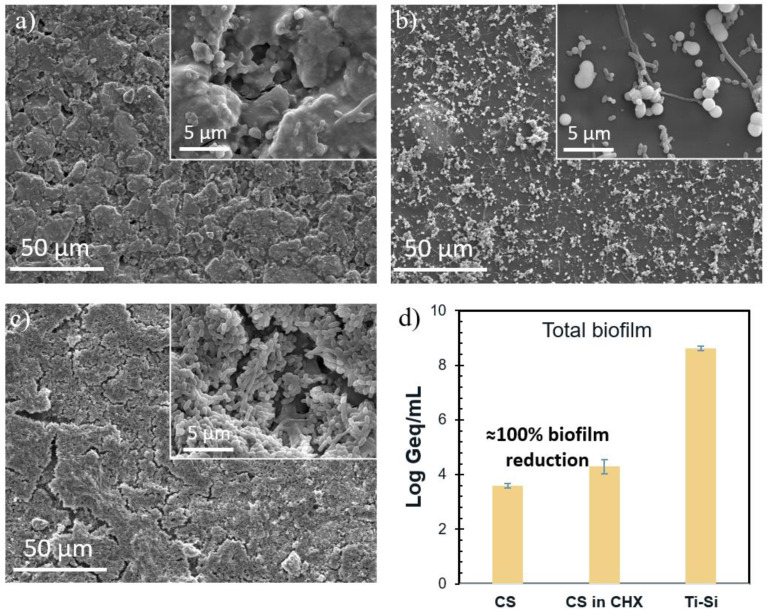
(**a**–**c**) SEM micrographs (scale bar of 50 µm with insets with scale bar of 5 µm) comparing the growth of bacterial strains on 1CS/G-coated Ti/SiO_2_ disk in (**a**) CHX and (**b**) PBS and (**c**) uncoated Ti/SiO_2_ disk indicating highly suppressed bacterial growth in (**a**,**b**) compared to the negative control (**c**). (**d**) Quantitative evaluation in genome equivalents/mL (Geq/mL) of the microorganisms using v-qPCR indicates a comparative count for samples used in (**a**,**b**) and significantly lower counts than negative control (**c**).

1CS/G-coated Ti/SiO_2_ samples were used for antimicrobial experiments (Figure 7) due to their pH-responsive and controlled CHX-releasing behavior, and three sample conditions were tested—a 1CS/G-coated Ti/SiO_2_ disk in CHX to measure the combined antibacterial efficacy of the CHX and CS coating, a 1CS/G-coated Ti/SiO_2_ disk in PBS to measure the antibacterial efficacy of the CS coating and an uncoated Ti/SiO_2_ disk in PBS as a negative control.

SEM micrographs show a high inhibition of bacterial growth on both 1CS/G-coated Ti/SiO_2_ samples in both CHX (Figure 7a) and PBS (Figure 7b) compared to the uncoated Ti/SiO_2_ sample in PBS (Figure 7c). Microorganism quantification using v-qPCR (Figure 7d) also indicates a ≥4 log10 reduction in the attached bacteria, corresponding to a 99.99% reduction in bacterial counts compared to the negative control for both tested samples.

## 4. Discussion

Dental-implant-related infections are on the rise due to the continuous exposure of the materials to hostile oral environments. Adhesion of bacteria on the implant surface leads to biofilm development. Delay in treating these biofilm-infected implants can cause peri-implant mucositis and in more severe cases peri-implantitis, which results in implant loss [8,32]. Systemic administration of antibiotics for the treatment of these infections is less effective and not sufficient to completely eradicate the formation of biofilm from the surface of implants as the resistance of bacteria inside a biofilm is a thousand-fold higher than planktonic bacteria [33]. Through our previous research and other works, it has been proven that localized drug delivery is more effective against the formation/treatment of a bacterial biofilm on an implant surface compared to systemic drug delivery [13,14,34,35,36]. It is possible to control the rate of drug release by varying the concentration of the feed solution and the pore size of the silica diffusion barrier [14]. In addition to these possibilities, the rate of drug release can also be controlled by capping silica pores.

In this research, a CS coating was applied onto the surface of Ti/SiO_2_ disks where the CS acted as a capping agent and the synergy between the controlled release and antibacterial properties of the CS coating with the Ti/SiO_2_ composite substrate was studied. The drug release was 33% lower for a 1CS/G-coated Ti/SiO_2_ substrate compared to uncoated Ti/SiO_2_ substrates. Thus, apart from preventing the growth of biofilm, cytotoxicity to the normal host tissues would also be avoided. This is evident from the drug-release experiments shown in Figure 5a where the Ti/SiO_2_/CS disks have significantly lower CHX release compared to the Ti/SiO_2_ disks. The presence of a CS layer acts as an additional barrier to the diffusion of CHX from the feed side to the release side. Similar results were obtained in a previous study by Beenken et al. where the presence of a chitosan coating on daptomycin-loaded calcium sulphate substrates reduced the burst release of the drug by a factor of 10 and prolonged the release period [16]. Although the daily CHX release rate for 1CS/G-coated Ti/SiO_2_ is still high compared to the biofilm inhibitory concentration (BIC) and minimum inhibitory concentration (MIC) values for biofilm inhibition studied by De Cremer et al., this study is a proof-of-concept, and the size of the substrates used is quite large compared to the actual dental implant where a similar concentration feed solution of CHX would result in a lower CHX release rate [14]. The barrier properties of the CS layer were confirmed from airflow measurements, which indicated that the CS-coated Ti/SiO_2_ disks were almost impervious to the incoming air, but it is essential to note that these measurements were taken in dry conditions. A limited drug release through these samples was noticeable due to the swelling properties of the CS film in an aqueous medium, which allowed the diffusion of CHX through the disks. Swelling of the CS film occurs as a means to balance the force arising from the swelling of CS polymer chains and the osmotic pressure which acts as a driving force to allow the solvent to pass through the swollen CS. This swelling phenomenon consists of three consecutive steps: (1) water molecule diffusion inside the polymer network, (2) relaxation of the hydrated CS polymer chains and (3) swelling of the CS polymer chains in the surrounding aqueous medium [37]. The swelling of GPTMS-crosslinked CS films is higher in an acidic medium compared to a neutral medium, as evident from the QCM-D measurements for S3 shown in Figure 4c,d. An increase in the magnitude of Δf corresponds to a rise in the mass of the coating due to water absorption [38]. This modulates the viscoelastic properties of the coating, as confirmed by the corresponding increase in ΔD. This is comparable with the observation found by Miras et al. where genipin crosslinked chitosan had higher swelling at pH 3 and shrunk at pH 6 [39]. This swelling happens due to the protonation of the free amine groups of the CS chain since the pH of the acidic medium is below the pKa value of CS, which is around 6.5 [40]. The intermolecular repulsion between the protonated molecules causes the swelling of the CS chains. The degree of swelling in an acidic environment is inversely proportional to the degree of crosslinking [39]. To prevent the film from disintegrating while swelling, GPTMS is used as a crosslinking agent to form an interconnecting network between the GPTMS and CS monomers. This is illustrated in Figure 4a comparing the swelling results between the non-crosslinked CS film and GTPMS-crosslinked CS films where the swelling in the neutral medium is nearly twice the latter. The crosslinking network improves the film’s mechanical properties and prevents the CS chains from dissociating in the aqueous medium [41]. The higher stability of the crosslinked CS film is also confirmed with the SDT results in Figure 4b where the earlier weight loss and higher heat evolution of the non-crosslinked CS film indicates the presence of more residual water inside the film compared to the crosslinked CS film. This indicates that the non-crosslinked film uptakes more water, leading to higher swelling than for the crosslinked CS film. As the temperature increases, the crosslinked CS film follows the trend of the non-crosslinked CS film, indicating delayed disintegration. At higher temperatures, the DSC signals are higher but the weight loss is lesser for the crosslinked CS film indicating the presence of a chitosan and silica (GPTMS) crosslinked structure. The increase in DSC signals could be due to the thermally stable silica which increases the thermal stability of the crosslinked CS film and reduces the thermal degradation compared to non-crosslinked CS film. A similar observation has been reported by Liu et al. [41].

CS coatings have been used in the literature to study their antimicrobial properties which are due to the electrostatic interaction between the positively charged amino groups of CS and the negatively charged components in the microbial membrane [42]. This antimicrobial property is evident below pH 6 [43]. Since some bacterial biofilms tend to create an acidic environment around them, CS is therefore an interesting biofilm-inhibiting material [17,18]. The hydroxyl groups of silica nanoparticles form covalent bonds with the hydroxyl groups of CS polymer chains, ensuring the robust adhesion of CS coating to the surface [44]. This is evidenced by the continuously suppressed CHX release and the presence of the coating within the Ti macropores in the SEM micrograph after CHX release, as indicated in Figure 6b. It is necessary to crosslink the CS chain to improve the durability of the coating, particularly in an acidic medium prevalent in bacterial biofilms. The oral environment is very hostile regarding pH (due to pathogens and food consumption) and mechanical loading (mastication action). The swelling and controlled release experiments in this study indicate that the crosslinked CS coating is highly durable as the Ti/SiO_2_/CS disks show suppressed release throughout the testing duration compared to the Ti/SiO_2_ disks. However, it would be of interest to study the stability of this composite system in different environments over time in future research. Experiments evaluating mechanical stability, surface aging, resistance to sterilization and storage could be of specific interest. Finally, to examine the antimicrobial activity, 1CS/G-coated Ti/SiO_2_ was chosen due to the pH-responsive and controlled release of CHX. As illustrated in the antimicrobial results in Figure 7, the 1CS/G-coated Ti/SiO_2_ disks in CHX and PBS showed almost similar reduced antimicrobial activity compared to the negative control where huge amounts of biofilm mass had grown on the sample surface. For the 1CS/G-coated Ti/SiO_2_ disk in PBS, the antimicrobial activity was solely because of the CS coating as the samples for this condition were freshly prepared and a homogeneous CS coating was formed, as seen in the respective SEM image [45]. In the case of the CHX-releasing 1CS/G-coated Ti/SiO_2_ disk samples, since the CS coating had aged over time and was anchored only to the silica nanoparticles inside the Ti macropores, the antimicrobial activity can be credited to the controlled CHX release through the Ti macropores and also to the CS regions on the samples. This sample condition better represents the long-term behavior of the samples, reflecting the gradual reduction of the CS coating on the Ti surface over time in the aqueous medium as it is not anchored to the Ti surface. On the contrary, the CS region anchored to the silica nanoparticles in the macropores of Ti via covalent bonds contributes to the controlled release of CHX and also demonstrates an antimicrobial tendency due to the charge interaction between CS and the bacterial membrane [42].

## 5. Conclusions

In order to incorporate controlled drug-release activity along with pH response and to study the antimicrobial efficacy of next-generation dental implants, we report here, for the first time, the synthesis and evaluation of GPTMS-crosslinked CS-coated Ti/SiO_2_ composite substrates with pH-triggered CHX-releasing abilities. Successful coating of crosslinked CS on Ti/SiO_2_ was confirmed using FT-IR spectroscopy, where bands specific to CS monomer were present for the GPTMS-crosslinked CS-coated Ti/SiO_2_ substrates, but not for the uncoated Ti/SiO_2_ substrate. Out of the different wt% of CS coating used for the CHX release experiments, the rate of CHX release through 1CS/G was around 33% lower compared to the uncoated Ti/SiO_2_ substrate, with 68.22 µg/mL (0.109 mM) and 101.69 µg/mL (0.162 mM) values, respectively. The rate of CHX release was higher in the acidic regions, which was indicated by an increase in the slope of the CHX-release profile indicating the pH response of the Ti/SiO_2_/CS substrate. This was confirmed by the QCM-D results on free-standing crosslinked CS films. The antimicrobial efficacy of Ti/SiO_2_/CS was also tested against a dual-species oral biofilm model of the biofilm-forming cariogenic pathogen *S. sobrinus* and the periodontal pathogen *F. nucleatum*. Both 1CS/G-coated Ti/SiO_2_ samples in CHX and PBS showed similar reduced numbers of bacterial growth compared to the uncoated Ti/SiO_2_ sample in PBS. The presence of a CS coating in fresh samples used for the PBS study demonstrates the good antimicrobial properties of CS when in immediate contact with bacterial strains. On the other hand, the samples having CS coating anchored only to the Ti macropores infiltrated with silica nanoparticles used for CHX-release testing (which also represent how the coating would transform over time) represents the long-term condition of the sample. Since both the sample conditions show similar behavior, this proves the superiority of the CHX-releasing 1CS/G-coated Ti/SiO_2_ sample as it can have a longer-lasting antimicrobial effect compared to the same sample without CHX.

## Data Availability

The data presented in this study are available on request from the corresponding author.
